# Predictors of Conversion to Multiple Sclerosis in Patients with Clinical Isolated Syndrome Using the 2010 Revised McDonald Criteria

**DOI:** 10.5402/2012/792192

**Published:** 2012-11-01

**Authors:** R. Alroughani, J. Al Hashel, S. Lamdhade, S. F. Ahmed

**Affiliations:** ^1^Division of Neurology, Amiri Hospital, Qurtoba 73767, Kuwait; ^2^Division of Neurology, Dasman Diabetes Institute, P.O. Box 1180, Dasman 15462, Kuwait; ^3^Department of Neurology, Ibn Sina Hospital, P.O. Box 25427, Safat 13115, Kuwait; ^4^Department of Medicine, Kuwait University, P.O. Box 24923, Safat 13110, Kuwait; ^5^Department of Neurology and Psychiatry, Minia University, P.O. Box 61519, Minia 61111, Egypt

## Abstract

*Background*. Clinically isolated syndrome (CIS) is the first neurologic episode of multiple sclerosis (MS). Magnetic resonance imaging (MRI) and clinical features are used to predict risk of conversion to MS. *Objectives*. The aim of this prospective study is to evaluate predictors of conversion of CIS to McDonald MS. *Method*. 97 patients with CIS have been followed for 2 years. Age of onset, gender, initial clinical presentation, and MRI brain and spine were assessed. The 2010 revised McDonald criteria were applied. *Results*. Fifty-nine patients (60.8%) with CIS converted to McDonald MS after 10.1 + 4.2 months. Thirty-seven (38.1%) of the convertors satisfied the diagnostic criteria based on the radiological parameters, while 21.7% sustained their second clinical events. A multivariate regression analysis revealed that high number of lesions in MRI (*P* = 0.001) and earlier age of onset (*P* = 0.043) predicted the conversion of CIS to McDonald MS. Gender (*P* = 0.5) and initial clinical presentation (optic pathway (*P* = 0.4), supratentorial (*P* = 0.91), brain stem/ cerebellum (*P* = 0.97), and spinal (*P* = 0.76)) were not statistically significant. *Conclusion*. Age of onset and MRI parameters can be used as predictors of CIS conversion to McDonald MS. Application of the 2010 revised McDonald criteria allows an earlier MS diagnosis.

## 1. Introduction

Clinically isolated syndrome (CIS) describes the first clinical episode of symptoms and signs suggestive of an inflammatory demyelinating disorder of the central nervous system (CNS)[1]. It is typically applied to adults aged 20–45 years who developed acute or subacute presentation of symptoms reaching a peak within one to three weeks. The attack should last for at least 24 hours and occurs in the absence of fever or infection, with no clinical features of encephalopathy [1, 2]. CIS is isolated in time (i.e., monophasic), and it is usually isolated in space (i.e., monofocal) with signs indicating a lesion in the optic pathway, spinal cord, brainstem, cerebellum, or rarely the cerebral hemisphere. However, some patients with a CIS have clinical evidence of dissemination in space (i.e., multifocal) affecting two or more locations [3].

Since CIS could be monophasic, some of the patients may not subsequently develop new symptoms or brain MRI lesions consistent with MS. Therefore, understanding the prognostic factors for MS after a CIS may help identifying patients who are at higher risk for developing clinically definite MS and have ongoing disease activity [4]. It has been shown that baseline MRI findings have the most predictive value in evaluating the risk of CIS conversion to MS [5]. The aim of our study is to assess the demographic, clinical and radiological prognostic factors in CIS patients and, to evaluate the risk of conversion to McDonald MS. 

## 2. Patients and Methods

Multiple sclerosis registry was established in Kuwait in 2010. This included different hospitals and centers including Amiri Hospital, Ibn Sina Hospital, and Dasman Research Center. All patients were followed on regular basis, and their clinical and radiological data were entered in the registry database. We conducted a prospective study to evaluate CIS patients over a period of 2 years. Patients with their first clinical event were included in the study within 3 months of their initial presentation. The revised 2010 McDonald criteria were used to define CIS [6]. Patients with progressive symptoms at onset, those who did not satisfy the CIS definition, or those who had symptoms or signs suggestive of other inflammatory disorders (e.g., acute disseminated encephalomyelitis (ADEM), neuromyelitis optica (NMO), or vasculitis) were excluded. 

Demographics (age, gender), clinical (age at onset, symptoms and signs at presentation, past and ongoing therapy), radiological and relevant CSF, and serological data were collected. Age at onset was dichotomized into 2 groups: patients aged <30 years and patients aged ≥30 years. The initial presentation was classified into four main localizations (supratentorial, optic pathway, brainstem/cerebellar, and spinal cord). Baseline and follow-up brain, cervical, and dorsal spine MRI scans were reviewed. Brain MRI typically included sagittal T1-weighted images, an axial FLAIR and/or T2-weighted images, and axial pre- and postgadolinium T1-weighted images. Technical parameters varied according to sites. The following parameters were assessed: total number of lesions with a diameter exceeding 3 mm, dissemination in space (DIS), and dissemination in time (DIT) criteria as adapted in the 2010 revised McDonald criteria [6]. Four different categories for the number of lesions were considered: 0 lesions; 1 to 3 lesions; 4 to 8 lesions; 9 or more lesions, as suggested by Tintoré et al. [7].

CIS patients were assessed at regular intervals (3–6 months), and MRI brain and spine were performed every 6 months. The diagnosis of multiple sclerosis was established if the patient sustained a second clinical neurological event or if the follow-up MRI revealed DIS and DIT according to the revised 2010 McDonald criteria. 

The following variables were evaluated using the multivariate analyses to predict CIS conversion to MS: patient's age, age at CIS onset, gender, symptoms at presentation (optic neuritis, brainstem/cerebellum, spinal cord, or supratentorial symptoms), and number of MRI lesions at onset. All analyses were performed using SPSS 19 for Windows. Simple descriptive statistical tests (mean and standard deviation) are used to describe the numerical values of the sample. The significance of the differences between the CIS group and the MS group was determined using a chi-squared test for nonparametric variables and an unpaired *t*-test to compare the parametric variables between the two groups. A multivariate logistic regression analysis was used to select the set of covariates and factors that were independently associated with outcome. A probability of (*P*) ≤0.05 is accepted as significant. The study received the approval of the local ethic committee, and all the patients signed the appropriate informed consents.

## 3. Results

Ninety-seven CIS patients were included in the study. 56.7% (*n* = 55) were females, and F : M sex ratio was 1.3 : 1. The mean age of patients and the mean age at onset were 26.88 ± 7.25 years and 25.02 ± 7.06 years, respectively. The CIS population was homogenous in terms of ethnicity. All except 9 patients (9.3%) initiated disease modifying therapies (DMTs) within 3 months of symptoms onset. 32% of patients presented with supratentorial symptoms at onset, while 30.9% of patients had spinal cord symptoms at onset. Brainstem/cerebellar and optic pathway involvements were seen in 17.5% and 23.7% of patients, respectively. Only 7.2% of patients presented with multifocal involvements as shown in Table 1. The median time between onset of symptoms and MRI performance was 2.24 weeks. With respect to baseline MRI parameters, 41.2% of patients had ≥9 T2 lesions, while only 2.1% of patients had a normal brain and spine MRI (Table 1).

Fifty-nine of CIS patients (60.8%) converted to MS at 10.0031 ± 4.2 months when followed for 2 years. 22.7% of the convertors had their clinical relapse, while 38.1% fulfilled the radiological McDonald criteria using dissemination in time at their follow-up MRI parameters (McDonald MR-MS).

Although females constituted the majority of the cohort, there was no difference between convertors and nonconvertors on the basis of gender. We found that patients <30 years of age were at more risk in converting to MS (50.5% versus 24.7%; *P* = 0.01), and the mean age at onset was significantly lower in CIS patients who converted to MS (21.62 ± 6.15 years versus 25.59 ± 6.89 years; *P* = 0.01) as shown in Table 2. The rate of conversion of CIS to MS was not significant when the initial presentations were assessed. Infratentorial presentations were frequent in our cohort but with relatively equal distribution among both groups. 

With respect to the radiological parameters, CIS patients who converted to MS had ≥9 lesions than the nonconvertor cohort (36.1% versus 5.2%; *P* = 0.002), while those with 1–3 lesions continued to behave as a monophasic condition (16.5% versus 3.4%; *P* = 0.001) (Table 2).

 Multivariate logistic analysis showed that younger age at onset (*P* < 0.03) and the presence of ≥9 lesions in MRI (*P* < 0.001) were independent predictors of CIS conversion to McDonald MS at 2 years. All other variables such as gender and initial clinical presentation (optic pathway, supratentorial, brain stem/cerebellum, and spinal symptoms) had no statistically predictive value as indicated in Table 3. 

## 4. Discussion

Clinically isolated syndrome is a term describing the first neurological episode that might be suggestive of multiple sclerosis. This may create a diagnostic and therapeutic dilemma given the difficulty in predicting who will be converting to MS. Over 80% of CIS patients with MRI lesions go on to develop MS, while approximately 20% have a self-limited process [8, 9]. Clinical findings in combination with brain/spine MRI and CSF analysis can be used in CIS patients to evaluate their risk for clinically definite MS (CDMS). MRI continues to be the most useful paraclinical tool at this stage. The application of the McDonald criteria allows an earlier MS diagnosis by using MRI parameters to define dissemination in time and space. Early detection and management of CIS is crucial to delay disability progression.

Occurrence of MS in geographic regions like Arab countries, other than the western hemisphere, is well recognized. Although the prevalence and incidence are low, recent reports suggest it is increasing. In Kuwait, the total incidence rate increased from 1.05/100,000 population in 1993 to 2.62/100,000 in 2000, which may be attributed to local environmental factors as suggested by Alshubaili et al. [10]. A similar increase in cases is noted in Iran [11]. Clinical patterns are generally similar to the “Western type.” Since there were no regional studies to assess the natural history of CIS, we decided to study the clinical and radiological patterns of our CIS subgroup and evaluate conversion to MS.

As our database is new and still accumulating chronic cases, we observed a high number of CIS ~25% in our MS registry [12]. In a study from Dubai, UAE, it was found that the proportion of CIS is very low, 2%, but it was derived from a relatively small cohort of 100 MS groups [13]. 

The majority of our CIS cohort converted to MS at 3–19 months (mean 10.12 ± 4.18 months). Time of conversion was similar among patients with mono- and multifocal presentations, and it was slightly shorter than in other studies. In Lyon, France, Confavreux et al. [14] studied the natural history of 1215 patients and they found that median time to second episode was 1.9 years while the mean time for conversion to MS was found to be 1.3–1.5 years when Ruet et al. assessed isolated spinal cord syndrome [15]. The earlier time of conversion in our study could be partly explained by the application of the 2010 revised McDonald criteria and short intervals of clinical and radiological assessments (3–6 months). Radiological conversion was seen in 37 out of 59 convertors indicting that the diagnosis could be established earlier using a preset radiological criteria. The previous (2005) McDonald criteria [16] identified twice as many patients with MS than Poser criteria [17] after 3–12 months of followup [18]. DIS criteria are simpler and more sensitive than previous criteria [19]. Rovira et al. found that although the sensitivity of DIT criterion using a single MRI scan was low (52.63%), MRI criterion remains a highly specific parameter that could improve the accuracy of early MS diagnosis in that group of patients with typical CIS [20]. 

Distribution of gender in our cohort (F : M ratio =1.3 : 1) is slightly lower than worldwide figures [21–24]. This could be partly explained by incomplete ascertainment of all CIS/MS patients since our registry has been recently established. Another factor is the social stigma in our community preventing females from entering their database into a national registry. 

The mean age at onset was significantly lower among the convertors, and the younger age (<30 years) was independently associated with a higher risk of developing a second event within 2 years of onset. Our results are consistent with previous studies which found that younger age is independently associated with the risk of a second relapse [25–27]. However, the mean age of MS onset was higher in other studies [28–32], which may reflect the difference in environmental and hereditary factors in our cohort in addition to the differences in the methodology and ascertainment.

 Although a large number of our convertors presented with spinal cord syndrome (19.3%) and the rate of conversion of CIS to MS was approximately 60%, a presentation with a spinal cord syndrome was not significant as a predictor. This is likely due to the small number of overall patients in each group. In patients with spinal cord CIS, conversion to MS has been reported to vary between 41% and 61% [33, 34]. The proportion of patients with brainstem syndromes who develop MS varies between 53% and 60% [35]. Miller et al. found that optic neuritis was associated with a lower risk of developing MS than other types of clinically isolated syndromes [36]. These differences in conversion rates might be explained on the basis of geographical variation in the natural history of MS, the lengths of followups, and the use of the specific diagnostic criteria. 

 Most of our CIS patients converted radiologically at their second and third MRI followups within the studied period. The applicability of DIT/DIS criteria has certainly influenced the high proportion of radiologically convertors in a two-year study. Chard et al. found that 15% of patients developed radiological definite MS using the previous McDonald criteria when CIS patients were prospectively followed for 6 years [37]. Gómez-Moreno et al. applied the revised 2010 McDonald criteria on 67 CIS patients with baseline MRI performed within the first 3 months after onset [19]. After at least 24 months, followup, the overall conversion rate was 74%. They concluded that the DIT criterion using a single MRI could improve the accuracy of early MS diagnosis in that group of patients with typical CIS and gadolinium-enhancing and nonenhancing lesions on their baseline scans [19]. 

The number of MRI lesions at baseline was a strong predictor to MS conversion in our CIS cohort. This was evident in 2 groups. Patients with 1–3 T2 lesions at baseline were at lower risk of conversion, while those who had ≥9 T2 lesion were at a higher risk. The number of T2 lesions at baseline MRI has been shown to correlate with the risk and time to development of CDMS [5, 7]. Tintoré et al. studied 156 CIS patients for a median of 7 years, and they found that patients with three to four Barkhof criteria had higher hazard ratio (HR) and hence a high conversion risk [7].

Using multivariate logistic analysis, two variables were found to be independent predictors of McDonald MS diagnosis at 2 years: younger age at onset and the presence of ≥9 lesions at baseline MRI. All other factors such as gender and initial clinical presentations had no predictive value that could be explained by low F : M sex ratio and the relatively small number of patients in each group when the initial presentations were divided. Our results are in agreement with other studies [25, 38]. West et al. studied 186 patients with CIS, non-white race and age <30 years at onset, who were associated in multivariate models with an increased risk of a second attack within a year of the CIS [39]. 

Our study has several limitations. First, our MS registry is still in its early stages and we have not captured most of the CIS/MS across all the geographical sites in the country. Second, we have not investigated other paraclinical predictors such as CSF oligoclonal band and evoked potentials. This was partly because the majority of patients either satisfied the McDonald criteria from the first MRI or elected to have a second MRI within 3–6 months prior to having a lumbar puncture. In the subgroup of patients who had less than 3 lesions on MRI, only 5 patients elected to have lumbar puncture and 3 of them had oligoclonal bands when their CSF was analyzed. Given the small number of this subgroup, we did not feel that analyzing this data would be of any clinical significance. On the other hand, our study is one of the first prospective studies in the region to assess the natural history of patients with MS and to evaluate the CIS conversion to MS. In addition, our CIS sample size is considered large when compared to regional MS studies. The applicability of the revised 2010 McDonald criteria in our study adds further importance to the radiological assessment of disease activity over time. 

In summary, our study indicated that younger age of patient, younger age of onset, and high (≥9) MRI lesion load are significant predictors in CIS patients when assessing the risk of conversion to MS. Application of the revised McDonald criteria 2010 allows an earlier MS diagnosis.

## Figures and Tables

**Table 1:** Baseline characteristics of CIS patients.

Variable	Number of patients (%)
Gender	
Female	55 (56.7%)
Male	42 (43.3%)

Age (mean ± SD)	26.88 ± 7.25

Age	
<30 years	70 (72.2%)
≥30 years	27 (27.8%)

Age at onset (mean ± SD)	25.02 ± 7.06

Age at onset	
<30 years	73 (75.3%)
≥30 years	24 (24.7%)

Clinical presentation	
Supratentorial	31 (32%)
Optic pathway	17 (17.5%)
Brainstem/cerebellar	23 (23.7%)
Spinal cord	30 (30.9%)
Multifocal	7 (7.2%)

Number of MRI lesions	
0	2 (2.1%)
1–3	18 (18.6%)
4–8	37 (38.1%)
≥9	40 (41.2%)

**Table 2:** Clinical and radiological predictors of CIS conversion to MS after 2 years.

Demographic data	CIS cohort	MS cohort	*P* value
	(*n* = 39)	(*n* = 58)	
Sex			
Female	21 (21.6%)	34 (35.1%)	0.4
Male	18 (18.6%)	24 (24.7%)	0.08

Age (mean ± SD)	30.21 ± 6.70	24.64 ± 6.77	0.01*

Age			
<30 years	18 (18.6%)	45 (46.4%)	0.002*
≥30	21 (21.6%)	13 (13.4%)	0.23

Age at onset (mean ± SD)	25.59 ± 6.89	21.62 ± 6.15	0.01*

Age at onset			
<30 years	24 (24.7%)	49 (50.5%)	0.01*
≥30	15 (15.5%)	9 (9.3%)	0.2

Clinical presentation			
Optic nerve	6 (6.2%)	11 (11.3%)	0.4
Brainstem/cerebellum	8 (8.2%)	15 (15.5%)	0.4
Spinal cord	11 (11.3%)	19 (19.3%)	0.5
Supratentorial	13 (13.4%)	18 (18.6%)	0.4

Number of lesions in MRI			
No lesion	(2.1%)	0 (0%)	
1–3 lesions	16 (16.5%)	2 (3.4%)	0.001*
4–8 lesions	16 (16.5%)	21 (21.7%)	0.2
≥9 lesions	5 (5.2%)	35 (36.1%)	0.002*

^∗^Statistically significant.

**Table 3:** Results of multivariate analysis of predictors of McDonald MS.

Predictors of McDonald MS	Odd ratio	Confidence interval (95%)	*P* value
Age at onset	0.38	0.15–0.97	0.043*

Gender	0.87	0.58–1.32	0.51

Clinical presentation			
Optic neuritis	1.31	0.66–2.63	0.44
Brainstem/cerebellum	1.01	0.48–2.16	0.97
Spinal cord	1.37	0.20–9.34	0.76
Supratentorial	1.12	0.11–11.82	0.91

Number of lesions in MRI			
1–3	2.14	0.18–25.13	0.54
4–8	9.18	1.12–75.10	0.034*
≥9	2.06	1.34–3.17	0.001*

^∗^Statistically significant.
